# Tubgcp3 Is Required for Retinal Progenitor Cell Proliferation During Zebrafish Development

**DOI:** 10.3389/fnmol.2019.00126

**Published:** 2019-05-24

**Authors:** Guobao Li, Daqing Jin, Tao P. Zhong

**Affiliations:** ^1^State Key Laboratory of Genetic Engineering, School of Life Sciences, Zhongshan Hospital, Fudan University, Shanghai, China; ^2^Shanghai Key Laboratory of Regulatory Biology, Institute of Molecular Medicine, School of Life Sciences, East China Normal University, Shanghai, China

**Keywords:** γ-TuSC, γ-TuRC, *tubgcp3*, cell cycle, ciliary marginal zone, zebrafish

## Abstract

The centrosomal protein γ-tubulin complex protein 3 (Tubgcp3/GCP3) is required for the assembly of γ-tubulin small complexes (γ-TuSCs) and γ-tubulin ring complexes (γ-TuRCs), which play critical roles in mitotic spindle formation during mitosis. However, its function in vertebrate embryonic development is unknown. Here, we generated the zebrafish *tubgcp3* mutants using the CRISPR/Cas9 system and found that the *tubgcp3* mutants exhibited the small eye phenotype. Tubgcp3 is required for the cell cycle progression of retinal progenitor cells (RPCs), and its depletion caused cell cycle arrest in the mitotic (M) phase. The M-phase arrested RPCs exhibited aberrant monopolar spindles and abnormal distributed centrioles and γ-tubulin. Moreover, these RPCs underwent apoptosis finally. Our study provides the *in vivo* model for the functional study of Tubgcp3 and sheds light on the roles of centrosomal γ-tubulin complexes in vertebrate development.

## Introduction

Centrosome, the major microtubule-organizing center (MTOC) in vertebrate cells, provides a major site for microtubule (MT) nucleation and plays key roles in bipolar spindle assembly during mitosis (Kellogg et al., 1994). A typical centrosome consists of a pair of centrioles surrounded by the pericentriolar matrix (PCM) (Bornens, 2002). Many proteins, including γ-tubulin (GCP1), γ-tubulin complex proteins (GCPs) and a large number of other centrosome-associated proteins, localize to PCM and are involved in the formation of mitotic spindles. Together with other GCPs, γ-tubulin forms two distinctly sized complexes: the γ-tubulin small complex (γ-TuSC) and the γ-tubulin ring complex (γ-TuRC). The γ-TuSC is a heterotetramer consisting of two copies of γ-tubulin and one copy each of GCP2 and GCP3/Tubgcp3. Seven γ-TuSCs with GCP4, GCP5, GCP6 and other accessory proteins assemble into the γ-TuRC, which facilitates MT nucleation by capping the minus ends of MTs and protecting them from depolymerization (Zheng et al., 1995). Centrosomes have critical roles in brain development, and mutations in genes encoding for centrosome-associated proteins have been shown to be genetically linked to neurodevelopmental disorders (Novorol et al., 2013; Chavali et al., 2014; Morris-Rosendahl and Kaindl, 2015; Buchwalter et al., 2016).

Many studies have shown that γ-TuSC is involved in MT nucleation and mitotic spindle assembly, and every component of γ-TuSC is indispensable for cell cycle progression (Oakley et al., 1990; Geissler et al., 1996; Knop et al., 1997; Yuba-Kubo et al., 2005; Xiong and Oakley, 2009; Pouchucq et al., 2018). In *Saccharomyces cerevisiae*, cells overexpressing the wild-type Spc98p/GCP3 or carrying the temperature-sensitive allele *spc98-1* arrest in mitosis with a defective spindle (Geissler et al., 1996). The duplication and separation of spindle pole body (SPB; yeast centrosome) are not affected in these cells. Disruption of gcpC/GCP3 in *Aspergillus nidulans* results in absent functional spindles and defective mitosis (Xiong and Oakley, 2009). In *Drosophila* mutants for disks*-degenerate 4* (*dd4*, which encodes GCP3), cells exhibit reduced density of spindle microtubules and delayed cell cycle progression from mitosis (Barbosa et al., 2000). Centriole duplication and separation are defective in some of the *dd4* mutant cells. In addition, γ-tubulin is missing from the spindle poles and becomes dispersed throughout the cell. Mitotic arrest has been observed in Hela, T98, and U87MG cell lines after depletion of GCP3 using siRNA (Draberova et al., 2015; Farache et al., 2016; Cota et al., 2017). However, Mikule et al. (2007) found that cells harboring wild-type p53 (RPE-1, BJ-1, HME-1, and HCT-116) arrest in G1 phase in a p53-dependent manner after being transfected with siRNA against GCP3. Given that U87MG cells expressing wild-type p53 show mitotic arrest, it remains unclear which types of cell cycle defects will happen in the GCP3-deleted cells with wild-type p53 gene. These different cell cycle defects may occur in a cell type-specific manner as these studies were carried out using different cell lines. Moreover, there have been no functional studies of GCP3 *in vivo* in a vertebrate system.

Among vertebrate models, the zebrafish provides many unique advantages over other rodents for gene functional study during early embryonic development. For example, owing to its external fertilization and rapid development, zebrafish early embryos can be easily visualized and manipulated. In addition, maternal gene products, synthesized during oogenesis and supplied to the egg, play essential roles in the earliest stages of zebrafish embryonic development (Pelegri, 2003). With disruption of any of the maternal-effect genes, the embryos continue to develop until the maternal supply is exhausted, which facilitates the functional study of these genes at relatively late developmental stages. The zebrafish retina is part of the central nervous system (CNS), and its neuroanatomy is well characterized. The ciliary marginal zone (CMZ), a proliferative region located at the periphery of the retina, provides an excellent model for the study of neurodevelopment (Novorol et al., 2013). The retina grows continuously throughout life and almost all retina neurogenesis comes from CMZ after the embryogenesis of retina is completed at 60 hpf (Marcus et al., 1999). The CMZ contains retinal stem cells (RSCs) and retinal progenitor cells (RPCs), exhibiting a peripheral-to-central arrangement pattern. The RSCs were located nearest to the periphery, the proliferative RPCs resided in the middle, and the post-mitotic RPC cells were positioned at the most central of the CMZ (Wehman et al., 2005; Cerveny et al., 2010; Valdivia et al., 2016; Wan et al., 2016). In CMZ, cell proliferation and differentiation are precisely coordinated for the growth of zebrafish eyes. Many cell proliferation and differentiation-associated genes have been studied using the zebrafish model (Wehman et al., 2005; Cerveny et al., 2010; Valdivia et al., 2016).

In this study, we explored the *in vivo* function of *tubgcp3* using the zebrafish model. We found that Tubgcp3 is essential for the development of zebrafish retina. Knockout of the *tubgcp3* gene resulted in the small eye phenotype exhibiting CMZ defects due to the abnormal cell cycle progression. Depletion of Tubgcp3 in RPCs caused mitotic arrest and apoptosis. Our findings reveal the critical roles of GCP3 in cell cycle progression, providing insights into the function of its associated complexes, γ-TuSC and γ-TuRC, in development, and establish a vertebrate model for further study.

## Materials and Methods

### Zebrafish Maintenance

Zebrafish (*Danio rerio*) were maintained and bred under standard conditions as previously described (Westerfield, 2000). Stages of embryonic development were determined according to their morphology (Westerfield, 2000). Embryos were treated with 0.003% 1-phenyl-2-thiourea (PTU) in egg water to prevent the production of pigment. The transgenic line *Tg(HuC:GFP)* was used in this study, in which the zebrafish brain is labeled with green fluorescent protein (GFP) (Park et al., 2000). All animal experiments were approved by the Institutional Animal Care and Use Committee, Fudan University.

### Generation of *tubgcp3* Mutants by the CRISPR/Cas9 System and Phenotypic Analysis

Disruption of *tubgcp3* transcript variant X1 (GenBank accession number XM_005170995) was performed using CRISPR/Cas9 technology. Cas9 nuclease target sites were designed using the ZiFiT Targeter online software. The target site in the exon 4 was selected and the corresponding sequence was 5′-GGTCCTCACAGAGGCTGAGCAGG-3′. Cas9 mRNA and guide RNA (gRNA) were synthesized using the mMESSAGE mMACHINE T7 Transcription Kit (AM1344, Invitrogen, United States) and MAXIscript T7 Transcription Kit (AM1322, Invitrogen, United States), respectively. Then, 300 pg Cas9 mRNA and 30 pg gRNA were co-injected into the one-cell stage zebrafish embryos to knockout the *tubgcp3* gene. The PCR primers for genotyping were as follows: forward (5′-ATTACGCAGAGGACCAAGA-3′) and reverse (5′-AAAATAGGATTTCATACAGGAACCCG-3′). The *tubgcp3* mutants were identified at 3 dpf by the developmental defects of the head and the arch. Heterozygous animals were incrossed to generate embryos for experimental analyses. Homozygous mutants were used as the experimental group and siblings as the control in this study.

For morphological measurements, embryos were anesthetized with 0.16 mg/mL Tricaine (A5040, Sigma-Aldrich) and embedded in 3% methylcellulose (M0387, Sigma-Aldrich). Body length was measured laterally in the rostral-caudal axis. Eye size was measured laterally along the longest diameter of the eye. Brain size was measured by calculating the dorsal area of the brain using the transgenic line *Tg(HuC:GFP*) (Park et al., 2000).

For cartilage staining, 5 dpf embryos were fixed in Bouin’s solution overnight. The fixed embryos were washed three times in 70% ethanol/0.1% ammonia, then washed two more times in 95% ethanol. The embryos were transferred into Alcian blue solution (80% ethanol/20% glacial acetic acid, 0.1% Alcian blue) and stained overnight. After staining, the embryos were gradually rehydrated in a decreasing ethanol series (60%, 40%, and 20%) and washed twice in phosphate-buffered saline (PBS). The embryos were incubated in 3% hydrogen peroxide/1% potassium hydroxide until the pigmentation was removed. After that, the samples were incubated in 0.05% trypsin in 30% sodium tetraborate until cleared. Then, the embryos were transferred into 70% glycerol for imaging.

### Semi-Quantitative RT-PCR and Plasmids

Total RNA was extracted from whole embryos at the indicated developmental stages using a TRIZOL reagent. cDNA synthesis was carried out using a TOYOBO RT-PCR kit (FSQ-301, TOYOBO, Japan) according to the manufacturer’s instructions. The expression profile of *tubgcp3* was analyzed using the following primers: *tubgcp3* forward (5′-AGAAGAGATGGCCGAGTGGG-3′) and reverse (5′-CGCCAGACGAGTTCTGAGTA-3′); β-*actin* forward (5′-CAGCCTTCCTTCCTGGGTAT-3′) and reverse (5′-GCCATACAGAGCAGAAGCCA-3′). cDNA encoding full-length *γ-tubulin* was amplified by PCR and inserted into pEGFP-N2 (Clontech, United States) using EcoR I and Xho I. cDNA encoding Full-length, N-terminal (Tubgcp3 1–551 aa) and C-terminal (Tubgcp3 552–906 aa) Tubgcp3 were amplified by PCR and cloned into pCMV-Myc (Clontech, United States) using EcoRI and BamHI.

### *In Situ* Hybridization Analyses

*In situ* hybridization was performed on 10 μm cryosections of zebrafish eyes. Briefly, zebrafish embryos were fixed in 4% paraformaldehyde overnight at 4°C. The next day, the fixed embryos were washed three times with PBS and dehydrated in 30% sucrose. Then, the embryos were embedded in OCT (Invitrogen) before being sectioned at 10 μm with a Leica cryostat (Leica CM1860, Leica Microsystems, Germany). For whole-mount *in situ* hybridization, the steps were performed as previously described (Thisse and Thisse, 2008).

To generate *in situ* probes, PCR products of *γ-tubulin*, *tubgcp2*, *tubgcp3*, *pcna*, *vsx2*, *col15a1b*, *ccnd1*, *atoh7*, and *cdkn1c* were amplified from cDNA and subcloned into pGEM-T Easy vector (A1360, Promega). Probe plasmids were digested with restriction enzyme to make DNA templates. Digoxigenin-labeled RNA probes were generated by *in vitro* transcription using the MAXIscript SP6/T7 Transcription Kit (AM1322, Invitrogen). *In situ* hybridization signals were detected by using NBT/BCIP (11681451001, Roche) for sections and BM-Purple (11442074001, Roche) for whole-mount embryos.

### BrdU Incorporation

To examine RPC proliferation, 66 hpf embryos were incubated in the thymidine analog 5-bromo-2-deoxyuridine (BrdU) (B5002, Sigma-Aldrich) at 10 mM/1% dimethyl sulfoxide (276855, Sigma-Aldrich) in embryo medium for 6 h at 28.5°C. Then, the embryos were rinsed twice with PBS before being fixed at 72 hpf. To examine RSC proliferation, 4 dpf embryos were incubated with 3 mM BrdU/1% dimethyl sulfoxide in embryo medium for 24 h before being fixed at 5 dpf.

### HE Staining

For Hematoxylin and Eosin (HE) staining, embryos were dehydrated using a graded ethanol series (50, 70, 90, 95, and 100%) before being transferred to xylene. Then, the embryos were embedded in paraffin wax and sectioned at 5 μm using a Leica Cryostat (Leica RM2235, Leica). HE staining was performed according to standard protocols.

### Immunofluorescence and TUNEL Assay

Embryos were fixed in 4% paraformaldehyde for 2 h at room temperature (RT) except for anti-γ-tubulin staining fixed in 4% formalin. Cryosections were prepared as described in the previous ISH section. Cryosections were boiled in 0.01 M citric acid (pH 6) for 30 min. After being washed three times in PBST (0.1% Triton X-100 and 0.1% Tween 20 in PBS), the sections were incubated in blocking solution (2% horse serum, 10% FBS, 0.1% Triton X-100, 0.1% Tween 20, 10% DMSO in PBS) for 60 min. The sections were then incubated with primary antibodies at 4°C overnight. Primary antibodies used in this study include rabbit anti-phosphohistone-H3 (Ser-10) (06-570, Millipore; 1:400), mouse anti-BrdU (11170376001, Roche; 1:300), rabbit anti-γH2AX (Ser-139) antibody (GTX127342, GeneTex; 1:300), mouse anti-PCNA (P8825, Sigma-Aldrich; 1:200), mouse anti-γ-Tubulin antibody (T5326, Sigma-Aldrich; 1:1000), mouse anti-α-Tubulin antibody (T9026, Sigma-Aldrich; 1:1000), mouse monoclonal anti-acetylated tubulin (T6793, Sigma-Aldrich; 1:1000), rabbit polyclonal anti-opsin (AB5404, Millipore; 1:1000), mouse monoclonal ZPR-1 (ab174435, Abcam; 1:500) and mouse monoclonal anti-phodopsin 4D2 (ab183359, Abcam; 1:1000). The sections were washed three times in PBST and incubated with secondary antibodies at RT for 2 h. Secondary antibodies were goat anti-mouse Alexa-Fluor-488 (A-11001, Invitrogen; 1:1000), goat anti-mouse Alexa-Fluor-594 (A-21135, Invitrogen; 1:1000), goat anti-mouse Alexa-Fluor-555 (A-21422, Invitrogen; 1:1000), goat anti-rabbit Alexa-Fluor-488 (A-11034, Invitrogen; 1:1000) and goat anti-rabbit Alexa-Fluor-594 (A-11037, Invitrogen; 1:1000). Sections were counterstained with 4′,6-diamidino-2-phenylindole (DAPI) (D3571, Invitrogen) when needed. The filamentous actin (F-actin) was stained with Alexa Fluor 488 phalloidin (A12379, Thermo Fisher; 1:100). For BrdU staining, sections were treated with 2M HCl for 1 h at RT before being incubated in blocking solution. For whole-mount immunostaining, the 4% paraformaldehyde fixed embryos were permeabilized by proteinase K digestion before being incubated in blocking solution.

To detect cell death, a TUNEL assay was performed on sections using the *In Situ* Cell Death Detection Kit (Roche) according to the manufactures’ instruction.

### Immunoprecipitation and Western Blotting

For immunoprecipitation, HEK293T cells were co-transfected with GFP-tagged zebrafish γ-tubulin and Myc-tagged zebrafish Tubgcp3 constructs using Lipofectamine 2000 Transfection Reagent (Invitrogen). After 48 h, transfected cells were lysed with lysis buffer (50 mM HEPES, pH 7.5, 150 mM NaCl, 1 mM MgCl_2_, 1 mM EGTA, 0.5% NP-40, protease inhibitor cocktail (04693132001, Roche)) and incubated on ice for 10 min. The lysate was obtained by centrifugation at 16,000 × *g* for 15 min at 4°C. The supernatants were incubated with anti-Myc antibody and protein A-Sepharose (GE Healthcare) at 4°C for 4 h. Then the precipitants were washed three times with lysis buffer before being boiled in SDS loading buffer for immunoblot. Zebrafish embryos (5 dpf) were collected, washed and homogenized in RIPA lysis buffer (P0013B, Beyotime Biotechnology, China) containing protease inhibitor cocktail (04693132001, Roche). The following primary antibodies were used: rabbit anti-Myc (A5598, Sigma-Aldrich; 1:1000), rabbit anti-GFP (50430-2-AP, ProteinTech; 1:2000), rabbit anti-TUBGCP3 (15719-1-AP, ProteinTech; 1:1000), mouse anti-β-Actin (CW0264M, CWBIO; 1:2000) and rabbit anti-β-Actin (GTX124388, GeneTex; 1:5000).

### Senescence-Associated β-Galactosidase (SA-β-gal) Assay

To detect cellular senescence, β-galactosidase assay was performed using the Senescence Cells Histochemical Staining Kit (CS0030, Sigma-Aldrich). Zebrafish embryos (5 dpf) were fixed in 0.2% glutaraldehyde at RT for 2 h. The fixed embryos were washed three times in PBS before being embedded in OCT (Invitrogen). Then, the embryos were sectioned at 10 μm for the assay.

### Imaging

The images of *in situ* hybridization, HE staining and SA-β-gal assay were captured using a Nikon ECLIPSE Ni microscope (Nikon) or Olympus microscope (IX83, Olympus). Immunostaining images were acquired using a Zeiss Axio Observer.Z1 microscope (Zeiss) or Nikon A1 confocal microscope (Nikon). Bright-field images of whole mount zebrafish embryos were taken using a Leica microscope (Leica M205FA, Leica).

### Statistical Analyses

To calculate cell proliferation and cell death in zebrafish retina, sections of the most central portion of the retina were selected for the analysis. The cell number was manually counted using ImageJ software within a defined region in the nasal part of the retina including the nasal CMZ (140 μm × 100 μm). Proliferating cells in embryo tails was counted within a defined region including ∼400 μm of the tails. Statistical analyses were performed with unpaired Student’s *t*-test using GraphPad Prism software. A *P*-value < 0.05 was considered statistically significant (^∗^*P* < 0.05, ^∗∗^*P* < 0.01). Data are expressed as mean + SEM.

## Results

### The Expression Pattern of Zebrafish *tubgcp3* During Early Development

**FIGURE 1:**
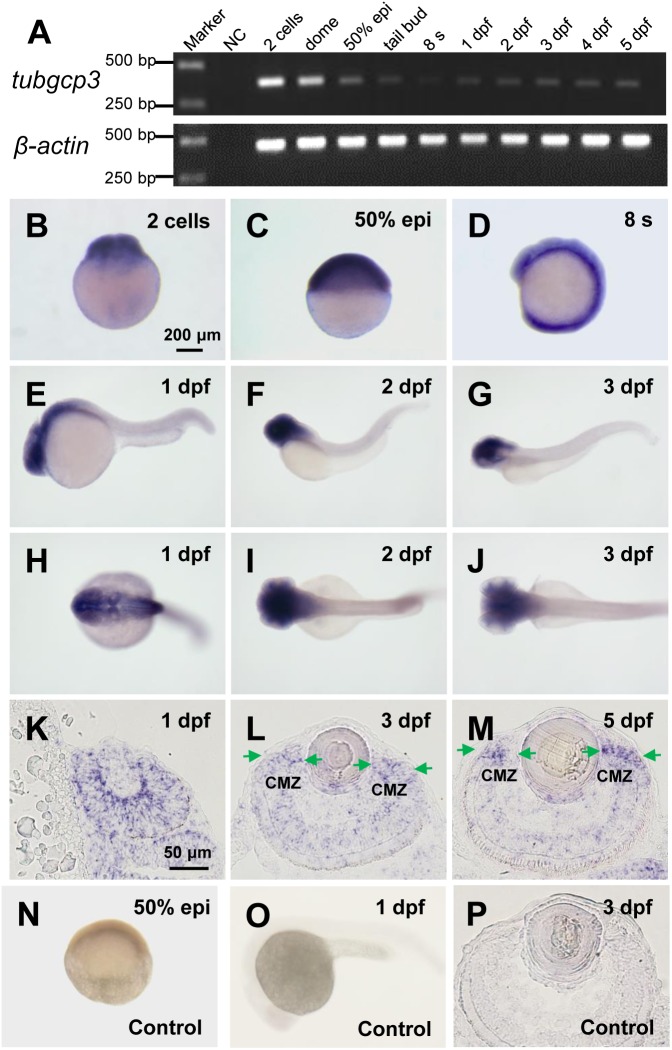
Expression of *tubgcp3* during zebrafish early development. **(A)** Semi-quantitative RT-PCR analyses showing the expression of zebrafish *tubgcp3* from 2-cell stage to 5 days post-fertilization (dpf). β-*actin* was used as the internal control. (**B–D**) Whole-mount *in situ* hybridization (WISH) displaying ubiquitous expression of *tubgcp3* at the two-cell stage **(B)**, 50%-epiboly **(C)** and 8-somite stage (8 s) **(D)**. **(E–J)** From 1 to 3 dpf, the expression of *tubgcp3* becomes concentrated at the head. **(K–M)** *In situ* hybridization (ISH) of zebrafish retinal cryosections exhibit the expression of *tubgcp3* throughout the whole retina at 1 dpf **(K)**. From 3 dpf, its expression is enriched at the ciliary marginal zone (CMZ) **(L,M)**. **(N–P)** No positive staining is detected with the sense probe. Arrows indicate the CMZ of the retina. Scale bars: 200 μm **(B–J,N,O)**; 50 μm **(K–M,P)**.

To analyze the roles of *tubgcp3* during zebrafish early development, we first examined the expression pattern of *tubgcp3* by RT-PCR (Figure 1A) and whole-mount *in situ* hybridization (WISH) (Figure 1B–J). The RT-PCR analyses showed *tubgcp3* transcripts were maternally deposited and continually expressed from 2-cell stage to 5 days post fertilization (dpf) during early embryonic development (Figure 1A). The expression of *tubgcp3* mRNA first decreased and then increased from 2-cell stage to 24 hpf, indicating the degradation of maternally deposited *tubgcp3* transcripts and the activation of zygotic transcripts during the maternal-to-zygotic transition (MZT). Consistent with RT-PCR results, WISH analyses showed that *tubgcp3* mRNA was detected at all developmental stages from cleavage stage to 3 dpf compared with the control group (Figure 1B–J,N,O). Furthermore, we found that the expression of *tubgcp3* was enriched in the head and eye as well as weak expression in other tissues of the embryos from 1 to 3 dpf (Figure 1B–J). To further characterize its expression in zebrafish retina, *in situ* hybridization (ISH) was performed on cryosections of the embryo eyes. In comparison with the sense probe as the control (Figure 1P), *tubgcp3* was expressed throughout the retina with a gradual enrichment at CMZ (Figure 1K–M). We also detected the expression of *tubgcp2* and *γ-tubulin*, which encodes another two subunits of γTuSC. Their expression was similar to that of *tubgcp3* (Supplementary Figures S1A–H). In addition, the expression of *pcna*, a proliferation marker, was confined in the CMZ from 3 to 5 dpf (Supplementary Figures S1I–L). These data indicate that *tubgcp3* may have a role in cell proliferation during zebrafish retinal development.

### Generation of Zebrafish *tubgcp3* Mutants and Morphological Analysis

**FIGURE 2:**
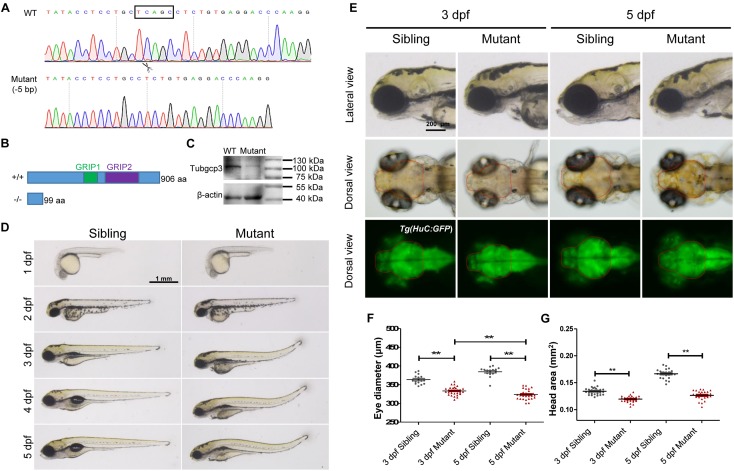
CRISPR/Cas9-mediated *tubgcp3* knockout results in zebrafish developmental defects. **(A)** Sanger sequencing displaying a 5-base pair (bp) deletion in *tubgcp3* gene in the zebrafish mutants. The black box indicates the deletion. **(B)** Predicted structure and amino acid sequence of the wild-type and the mutant alleles of Tubgcp3. The 5-bp deletion in *tubgcp3* gene was predicted to generate a 99 amino acid (aa)-truncated Tubgcp3 protein without the GRIP1 domain (green box) and GRIP2 domain (purple box). **(C)** Western blotting analysis showing Tubgcp3 protein decreased in *tubgcp3* mutant embryos at 5 dpf. **(D)** Whole-mount lateral views of the *tubgcp3* mutant and sibling embryos at the indicated developmental stages. **(E)** Higher magnification of the lateral and dorsal views of zebrafish heads from siblings and *tubgcp3* mutants at 3 and 5 dpf. The brain size is measured based on the fluorescent area in the head of *Tg(HuC:GFP)*. Red dotted lines indicate the brain area of the embryos used for analysis. **(F,G)** Scatter plot of eye and head size from wild-type siblings and *tubgcp3* mutants at 3 and 5 dpf. Data are from 29 embryos for each group. Student’s *t*-test: ^∗∗^*P* < 0.01. Scale bars: 1 mm **(D)**; 200 μm **(E)**.

To investigate the roles of *tubgcp3* during zebrafish embryonic development, we established *tubgcp3* knockout zebrafish lines using the CRISPR/Cas9 system. The CRISPR/Cas9 target site was designed in the exon 4 of the *tubgcp3* gene. Finally, two mutant alleles with 5-base pair (bp) deletion and 11-bp insertion were identified (Figure 2A and data not shown). Since the two mutant lines exhibited the same phenotypes, we used the 5-bp deletion line for further research. The 5-bp deletion was predicted to lead to a premature termination in Tubgcp3 protein translation and yield a 99-amino acid (aa)-truncated Tubgcp3 protein absent of the two conversed GRIP domains (Figure 2B). *tubgcp3* transcripts were detected in the *tubgcp3* mutant retina (Supplementary Figure S2A), which suggested that the mutated *tubgcp3* mRNA escaped non-sense-mediated mRNA decay (NMD). In addition, sequencing analyses confirmed that the 5-bp deletion transcripts existed in *tubgcp3* mutants (Supplementary Figure S2B). Furthermore, the full-length Tubgcp3 protein was significantly decreased in mutant embryos (Figure 2C). The residual full-length Tubgcp3 in *tubgcp3* mutants maybe attributed to the maternal supplied Tubgcp3 that was unusually stable in its associated complexes or depleted at a very low rate in embryos. All these results indicate a loss-of-function mutation in *tubgcp3* gene.

The *tubgcp3* mutant embryos were indistinguishable from their wild-type siblings before 2 dpf. From 3 to 5 dpf, the mutants exhibited a progressed MCPH (microcephaly)-like phenotype with reduced brain and eye size (Figure 2D,F). We determined the small brain phenotype using transgenic *Tg(HuC:GFP)*, in which GFP expression is controlled by the promoter of neuronal gene *HuC* (Park et al., 2000). We found that the brain size was reduced about 10% in the *tubgcp3* mutants compared to the wild-type siblings at 3 dpf (Figure 2G). The brain size reduction in *tubgcp3* mutants became more significant (about 24% reduction) when measured at 5 dpf (Figure 2G). Notably, the eyes became smaller in the *tubgcp3* mutants when compared with wild-type siblings from 3 dpf to 5 dpf (Figure 2E,F). Other defects including dorsal tail curvature (Figure 2D), uninflated swim bladder (Figure 2D), body length reduction (Supplementary Figure S3A) and jaw malformation (Supplementary Figure S3B) were also visible in *tubgcp3* mutants. The developmental defects were gradually more serious, and the mutants died around 10–14 dpf.

### The *tubgcp3* Mutants Exhibit Abnormal CMZ

**FIGURE 3:**
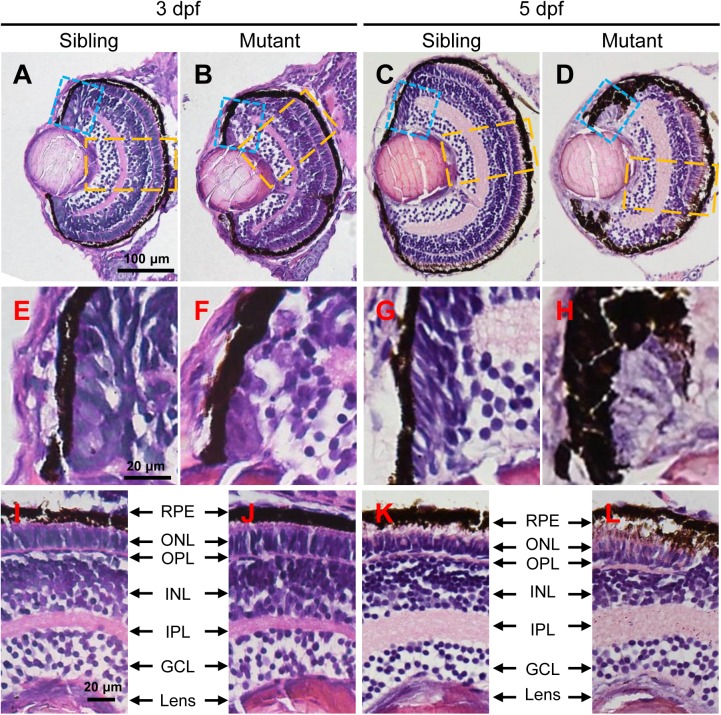
The *tubgcp3* mutants exhibit developmental defects in the CMZ of the retina. **(A–D)** Hematoxylin and Eosin (HE) staining displaying significant defects in the CMZ of the *tubgcp3* mutant retinae at 3 dpf and 5 dpf. **(E–H)** Higher-magnification images of the CMZ in blue dotted rectangles in **(A–D)**. **(I–L)** Higher-magnification image of the orange dotted rectangles in **(A–D)**. The *tubgcp3* mutants exhibit normal retinal laminar structures in the central retina. GCL, ganglion cell layer; IPL, inner plexiform layer; INL, inner nuclear layer; OPL, outer plexiform layer; ONL, outer nuclear layer; RPE, retinal pigment epithelium. Scale bars: 100 μm **(A–D)**; 20 μm **(E–L)**.

In order to investigate the eye defects in *tubgcp3* mutants in detail, we performed Hematoxylin and Eosin (HE) staining on transverse eye paraffin sections. The *tubgcp3* mutant retina showed defective CMZ with disorderly cells when compared to wild-type siblings at 3 dpf (Figure 3A,B,E,F). Notably, these abnormal cells disappeared from the CMZ at 5 dpf (Figure 3C,D,G,H). However, the laminar structure of central retina appeared normal in *tubgcp3* mutants at 3 dpf and 5 dpf (Figure 3I–L), including the ganglion cell layer (GCL), inner plexiform layer (IPL), inner nuclear layer (INL), outer plexiform layer (OPL), outer nuclear layer (ONL) and retinal pigment epithelium (RPE).

**FIGURE 4:**
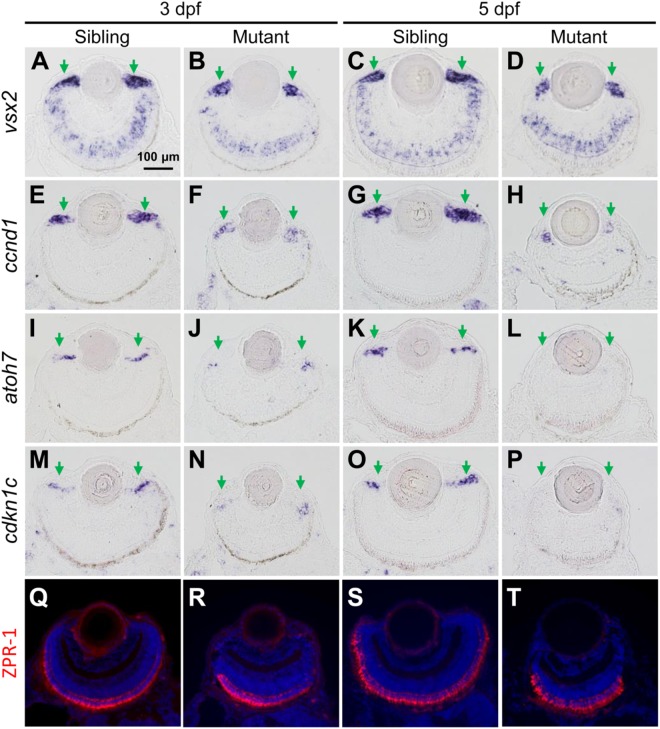
The *tubgcp3* mutant embryos exhibit decreased expression of proliferation and differentiation markers in CMZ cells. **(A–D)** ISH analyses show that the expression of *vsx2* is normal in the central retina but significantly reduced in the CMZ in *tubgcp3* mutant retinae at 3 dpf and 5 dpf. **(E–H)** ISH analyses exhibit that *ccnd1* is highly expressed in wild-type sibling CMZ **(M,O)** but clearly reduced in *tubgcp3* mutant CMZ **(N,P)** at 3 dpf and 5 dpf. **(I–P)** *atoh7* and *cdkn1c* (associated with retinal cell differentiation) are expressed in the central CMZ of wild-type sibling retina but significantly reduced in *tubgcp3* mutant CMZ at 3 dpf and almost disappeared at 5 dpf. **(Q–T)** Immunostaining analyses displaying normal ZPR-1 staining (green/red double cone photoreceptors marker) in the central area of the *tubgcp3* mutant retina. Arrows indicate the CMZ of the retina. Scale bars: 100 μm (**A–T**).

Ciliary marginal zone is responsible for adding new neurons to the continuously growing retina in zebrafish larvae, which consists of retinal stem cells (RSCs) and retinal progenitor cells (RPCs) (Wehman et al., 2005; Cerveny et al., 2010; Valdivia et al., 2016; Wan et al., 2016). *vsx2* encodes a homeodomain transcription factor, which is expressed in CMZ cells, Müller glia and a subpopulation of bipolar cells (Vitorino et al., 2009). The expression region of *vsx2* was reduced in *tubgcp3* mutant CMZ from 3 to 5 dpf (Figure 4A–D), which was consistent with the development defects detected by HE staining (Figure 3E–H). *col15a1b* expressed at the most periphery of CMZ, where it was considered to be a stem-cell niche (the location of RSCs) (Pujic et al., 2006; Cerveny et al., 2010; Gonzalez-Nunez et al., 2010; Valdivia et al., 2016). Its expression seems to be unaffected in the *tubgcp3* mutants (Supplementary Figure S4A). *ccnd1*, a marker for proliferating cells, encodes Cyclin D1 (CCND1) which is required for G1-S transition (Cerveny et al., 2010; Valdivia et al., 2016). The expression of *ccnd1* was significantly reduced in *tubgcp3* mutant CMZ (Figure 4E–H). *atoh7* encodes ATH5, a member of bHLH transcription factor, and is involved in the differentiation of ganglion cells (Masai et al., 2000; Cerveny et al., 2010; Valdivia et al., 2016). *cdkn1c* encodes the p57^cip/kip^, a cyclin-dependent kinase inhibitor, which is required for many retinal cells to exit from the cell cycle before their further differentiation (Ohnuma et al., 1999; Shkumatava and Neumann, 2005; Cerveny et al., 2010; Valdivia et al., 2016). The expression of *atoh7* and *cdkn1c* was significantly reduced in *tubgcp3* mutant CMZ at 3 dpf and almost disappeared at 5 dpf (Figure 4I–P). These results reveal that the *tubgcp3* mutants suffer from a significant reduction in proliferating and differentiating in CMZ cells.

In addition, different retinal cell types, including Müller glia cells (expressing *vsx2*) (Figure 4A–D), a subset of bipolar cells (expressing *vsx2*) (Figure 4A–D), cones (labeled with anti-ZPR1 for double cone photoreceptors and anti-M-opsin for cone outer segments) (Figure 4Q–T and Supplementary Figure S4B) and rods (labeled with anti-4D2 for rod outer segments) (Supplementary Figure S4B), appeared normal in the *tubgcp3* mutants. Moreover, cilia in photoreceptor cells (labeled with anti-acetylated α-tubulin) and retinal laminar structures, including the inner segment, inter plexiform layer (IPL), the outer limiting membrane (OLM) and the outer plexiform layer (OPL) (labeled with phalloidin) (Supplementary Figure S4B and data not shown), were also normal in *tubgcp3* mutants. These results suggest that these differentiated cells and structures are unaffected in the central reitna of the *tubgcp3* mutants.

### Loss of Tubgcp3 in CMZ Cells Causes M-Phase Arrest

Given the reduction of cell proliferation and differentiation in the *tubgcp3* mutant CMZ (Figure 4E–P), we first examined whether cell cycle progression was defective in mutant retinae using BrdU incorporation as an S phase marker and Phospho-Histone H3 (PH3) as a mitotic marker. Considering the length of the cell cycle in the CMZ at early larval stages is about 6 to 8 h (Li et al., 2000; Wehman et al., 2007), zebrafish embryos were incubated with BrdU from 66 to 72 hpf and then subjected to section and cell cycle analysis. Cycling RPCs would be labeled by BrdU except those that had passed S phase and would exit cell cycle and undergo differentiation.

**FIGURE 5:**
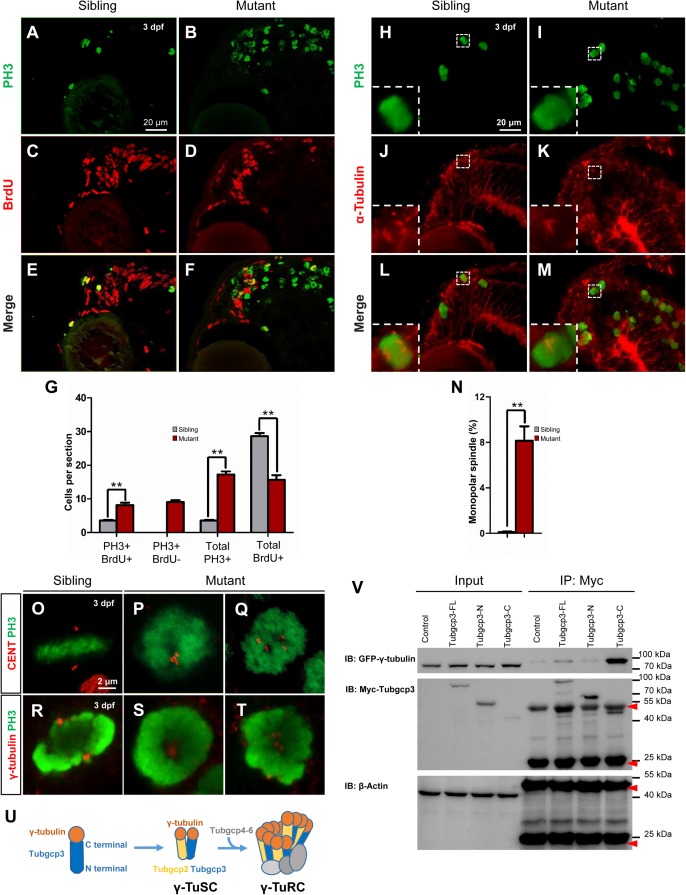
The *tubgcp3* mutant CMZ cells arrest in M-phase showing monopolar spindles and abnormal distributed centrioles and γ-tubulin. **(A–F)** Immunostaining analysis of cell proliferation in zebrafish retina at 3 dpf using DNA replication marker (BrdU, red) and mitotic marker (PH3, green). Embryos are incubated with BrdU for 6 h before being collected at 72 hpf for the analysis. Almost all cells in wild-type sibling CMZ are BrdU+ with several PH3+ cells among them **(A,C,E)**. In the (*tubgcp3* mutant retina, PH3+ cells are significantly increased **(B,F)**, but BrdU+ cells are markedly decreased **(D,F)**. Note that PH3+ BrdU- cells are detected in the *tubgcp3* mutant retina **(F)** but absent in the wild-type sibling **(E)**. **(G)** Bar chart analyses depicting quantification of BrdU- and PH3-labeled cells in wild-type sibling and *tubgcp3* mutant retinae. Data are mean + SEM from 50 retinal sections for each group. Student’s *t*-test: ^∗∗^*P* < 0.01. **(H–M)** Immunostaining of 3 dpf retinal cryosections with anti-α-tubulin (red) and anti-PH3 (green) displaying bipolar spindles formed in mitotic cells in wild-type siblings **(H,J,L)**. In the *tubgcp3* mutant retina, many mitotic RPCs exhibit monopolar spindles **(I,K,M)**. Insets indicate high-magnification images of mitotic RPCs in rectangles in **(H–M)**. **(N)** Bar charts depicting quantification of mitotic cells with monopolar spindles in wild-type sibling (0.12 per section, *n* = 43 sections) and the *tubgcp3* mutant retinae (8.14 per section, *n* = 36 sections). **(O–Q)** Immunostaining analyses displaying a pair of centrioles at each pole of the bipolar spindle in mitotic cells in wild-type sibling CMZ **(O)**. In the *tubgcp3* mutant retinae, centrioles are distributed at the center of the M-phase arrested cells (57.6%, *n* = 59 M-phase arrested cells) **(P)** or randomly scatter in these cells (42.4%, *n* = 59 M-phase arrested cells) **(Q)**. **(R–T)** Immunostaining analyses exhibiting γ-tubulin at the spindle poles in mitotic cells in wild-type sibling **(R)**. In *tubgcp3* mutant retinae, γ-tubulin localizes at the center of the M-phase arrested cells, showing a single focus (62.5%, *n* = 80 M-phase arrested cells) **(S)** or scattered foci (37.5%, *n* = 80 M-phase arrested cells) **(T)**. **(U)** Schematic representation of the structure of γ-TuSC and γ-TuRC. **(V)** Co-immunoprecipitation (IP) assays showing Tubgcp3 interacts with γ-tubulin through its C terminal domain. HEK293T cells were transfected with plasmids to express GFP-tagged zebrafish γ-tubulin and Myc-tagged zebrafish Tubgcp3 fragments, including full length (1–906 aa) Tubgcp3, N terminal (1–551 aa) Tubgcp3 and C terminal (552–906 aa) Tubgcp3. Then the cell samples were performed by immunoprecipitation with anti-Myc antibody and analyzed by immunoblotting (IB) with anti-Myc and anti-GFP antibodies. β-Actin was used as the loading control. Arrowheads indicate the IgG heavy chain (∼50 kDa) and IgG light chain (∼25 kDa). Scale bars: 20 μm **(A–F)**; 20 μm **(H–M)**; 2 μm **(O–T)**.

We compared expression patterns of BrdU and PH3 between the *tubgcp3* mutants and wild-type siblings. In wild-type siblings, the entire CMZ was labeled with BrdU with about three PH3+ cells scattered along the CMZ (Figure 5A,C,E,G). In the *tubgcp3* mutants, there was a significantly increased number of PH3+ cells in CMZ and its adjacent regions (Figure 5B,F,G). We noticed that a portion of these PH3+ cells were BrdU negative (Figure 5F). In contrast, there were no PH3+ BrdU- cells in wild-type siblings (Figure 5E). In addition, BrdU+ cells were significantly reduced in mutant CMZ (Figure 5D,F,G). The PH3+ BrdU- cells in *tubgcp3* mutants had passed S phase when we performed BrdU treatment, and they then went into mitosis as they were stained by PH3. However, these cells were still PH3+ after the 6 h BrdU treatment, which indicates that they were still in the mitotic (M) phase. Since G2 and M phase are shorter than 6 h, the results suggest that these RPCs were arrested in M-phase. Moreover, there were also more BrdU+ PH3+ cells in the *tubgcp3* mutants than in wild-type siblings (Figure 5E,F,G), suggesting that some of the cycling RPCs were arrested in M-phase after they passed the S phase. When whole-mount immunostaining was carried out under the same BrdU treatment condition, we observed similar results that a large number of PH3+ BrdU- cells existed in the *tubgcp3* mutant tails while they absent in wild-type siblings (Supplementary Figures S5A,B). These results suggest that Tubgcp3 is required for RPCs to go through mitosis.

Given the mitotic arrested cells detected in the *tubgcp3* mutant retinae, we analyzed the spindle formation by immunostaining using anti-α-tubulin and anti-PH3 antibodies. In wild-type siblings, bipolar mitotic spindles were formed in mitotic cells (Figure 5J,L). In contrast, abnormal monopolar spindles were observed in the mitotic arrested cells in *tubgcp3* mutant retina (Figure 5K,M,N). The microtubules arrayed radially in the center and the condensed chromosomes located at the periphery in these cells (Figure 5I,K,M). These results suggest that depletion of Tubgcp3 causes a defect in mitotic spindle formation in RPCs, resulting in mitotic arrest in these cells.

### Tubgcp3 Deficiency Impairs Centrioles Distribution

Since centriole duplication and segregation are essential for bipolar spindle formation during mitosis, we examined whether these processes were affected in the *tubgcp3* mutant retinal cells using anti-centrin (a marker for centriole) and anti-PH3. In wild-type siblings, the mitotic cells had a pair of centrioles (two centrioles per centrosome) at each spindle pole (Figure 5O). In the *tubgcp3* mutants, the M-phase arrested cells had four centriole dots like the wild-type siblings (Figure 5P,Q). However, the distribution of centrioles was abnormal. They were located at the center of the cell (Figure 5P) or randomly scattered in the cell (Figure 5Q). These data suggest that the centrioles duplicate normally but fail to separate correctly in mitotic arrested RPCs in the *tubgcp3* mutants.

Previous work has reported that Tubgcp3 interacts with γ-tubulin to form γ-TuRC complexes, which are located at the centrosome during mitosis (Pereira et al., 1998; Kollman et al., 2010; Figure 5U). We next detected whether the distribution of γ-tubulin was affected after Tubgcp3 depletion. Our immunostaining results showed that γ-tubulin located at each spindle pole during mitosis in wild-type siblings (Figure 5R). In contrast, the distribution of γ-tubulin was abnormal in the mitotic arrested RPCs in *tubgcp3* mutants. Most of the arrested cells that displayed γ-tubulin focus in the center of the cells (Figure 5S). Others showed scattered γ-tubulin staining patterns (Figure 5T). In order to test whether the interaction between Tubgcp3 and γ-tubulin is conserved in zebrafish, we performed co-Immunoprecipitation (co-IP) assays using HEK293 cells by co-transfecting Myc-tagged zebrafish Tubgcp3 and GFP-tagged zebrafish γ-tubulin. Co-IP assays showed that Tubgcp3 binds to γ-tubulin through its C-terminal domain, which is consistent with previous studies (Figure 5V). Taken together, our data suggest that depletion of Tubgcp3 does not affect centriole duplication, but results in abnormal distribution of centrioles and γ-tubulin.

### Tupgcp3-Deficient Cells Undergo Apoptosis and Senescence

**FIGURE 6:**
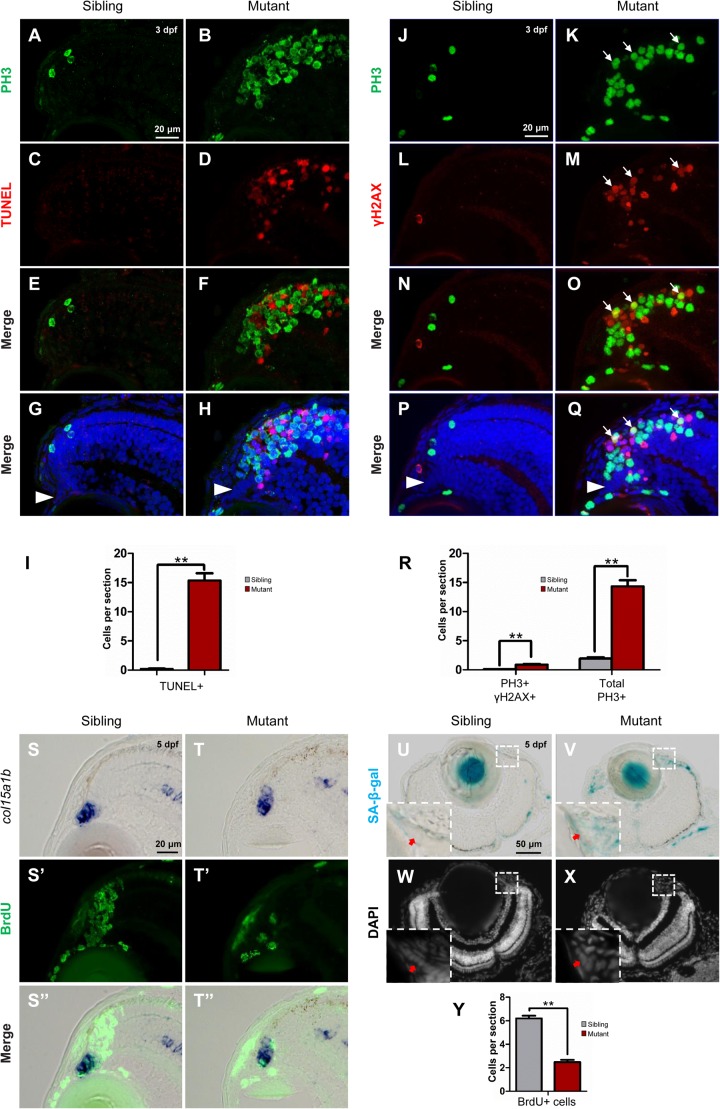
Tupgcp3 deficient cells undergo apoptosis and senescence. **(A–H)** Immunostaining analyses displaying significantly increased TUNEL+ cells in the *tubgcp3* mutant retina **(D,F,H)** at 3 dpf compared to wild-type sibling **(C,E,G)**. Note the increased PH3+ cells in the *tubgcp3* mutant retina do not co-localize with the TUNEL+ cells **(F,H)**. **(I)** Bar chart analyses depicting quantification of TUNEL+ cells in wild-type sibling and the *tubgcp3* mutant retinae. **(J–Q)** Immunostaining analyses exhibiting markedly increased γ-H2AX+ cells in the *tubgcp3* mutant retina **(M,O,Q)** at 3 dpf compared to wild-type sibling **(L,N,P)**. Note that some of the (increased PH3+ cells overlap with the γ-H2AX+ cells in the *tubgcp3* mutant **(O,Q)**. Arrows mark the PH3 and γ-H2AX double positive cells in the *tubgcp3* mutant retina. Arrowheads indicate the location of RSCs in the CMZ of the retina. **(R)** Bar chart analyses depicting quantification of γ-H2AX+ and PH3+ cells in wild-type sibling and the *tubgcp3* mutant retinae. Data are mean + SEM from 30 sections for each group. Student’s *t*-test: ^∗∗^*P* < 0.01. **(S–T”)** ISH and immunostaining analysis of cell proliferation at the extreme periphery of CMZ at 5 dpf. Zebrafish embryos were incubated in BrdU for 24 h before collected at 5 dpf for the double staining assay. In the wild-type sibling, there are many BrdU+ cells in *col15a1b*-labeled region **(S,S’,S”)**. In contrast, BrdU+ cells are significantly decreased in this region in the *tubgcp3* mutant CMZ **(T,T’,T”)**. **(U–X)** Senescence-associated β-galactosidase (SA-β-gal) staining exhibiting increased β-galactosidase activity at the CMZ in the *tubgcp3* mutant **(V)** compared to the wild-type sibling **(U)**. Nuclei are stained with DAPI **(W,X)**. Insets indicate high-magnification images of the peripheral edge of CMZ in rectangles in **(U–X)**. **(Y)** Bar chart analyses depicting quantification of BrdU+ cells in the *col15a1b*-labeled region in wild-type sibling and the *tubgcp3* mutant CMZ. Data are mean + SEM from 36 sections for each group. Student’s *t*-test: ^∗∗^*P* < 0.01. Scale bars: 20 μm **(A–H)**; 20 μm **(J–Q)**; 20 μm **(S–T”)**; 50 μm **(U–X)**.

Given our HE staining results showed disappearance of cells in the *tubgcp3* mutant CMZ from 3 to 5 dpf (Figure 3A–H), we next investigated whether the mitotic arrest RPCs underwent apoptosis using TUNEL assay. There were almost no TUNEL+ cells in wild-type siblings (Figure 6C,E,G,I). In contrast, TUNEL+ cells were significantly increased in *tubgcp3* mutant CMZ and the adjacent regions (Figure 6D,F,H,I) at 3 dpf. However, the TUNEL+ cells and PH3+ cells did not co-localize in the mutants (Figure 6F,H). This may be due to the fact that PH3 expression was absent in late-stage apoptotic cells (Huang et al., 2006). Next, we performed immunostaining using γ-H2AX, an early stage apoptotic marker (Rogakou et al., 2000), together with PH3. There was a significant increase in the number of γ-H2AX + cells in the *tubgcp3* mutant retina at 3 dpf (Figure 6M,O,Q,R), compared to wild-type siblings (Figure 6L,N,P,R). Moreover, some of the γ-H2AX+ cells overlapped with PH3+ cells (Figure 6O,Q). These results suggest that the M-phase arrested RPCs undergo apoptosis in the *tubgcp3* mutants.

In addition, RSCs [marked by *col15a1b* (Pujic et al., 2006; Cerveny et al., 2010; Gonzalez-Nunez et al., 2010; Valdivia et al., 2016)] still could be detected at the most peripheral CMZ (close to the lens) in the *tubgcp3* mutant retina at 5 dpf (Supplementary Figure S4A). Apoptosis signal was also absent in this region at 3 dpf (Figure 6H,Q) and 5 dpf (Supplementary Figure S6). We next want to detect cell proliferation at the peripheral CMZ. Embryos were incubated with BrdU for 24 h before being fixed at 5 dpf for the assay. In this region, BrdU+ cells were significantly decreased in the *tubgcp3* mutants compared to wild-type siblings (Figure 6S–T”,Y). As the proliferation of RSCs occurs at the peripheral CMZ, we speculated that RSC proliferation might be affected in the *tubgcp3* mutant CMZ. Previous studies have reported that centrosome dysfunction caused by centrosomal protein depletion could induce cellular senescence (Manning and Kumar, 2010; Schmidt et al., 2010; Hossain and Tsang, 2013). Consistent with these reports, we found that the expression of senescence-associated β-galactosidase (SA-β-gal), a widely used senescent marker (Dimri et al., 1995), was significantly increased at the peripheral CMZ in the *tubgcp3* mutant CMZ compared to the wild-type siblings (Figure 6U–X). These results indicate that depletion of Tubgcp3 caused cellular senescence at the peripheral CMZ that contains RSCs.

## Discussion

In this study, we generated the zebrafish *tubgcp3* mutant and investigated the function of *tubgcp3* in eye development. Our data reveal that Tubgcp3 plays important roles in cell cycle progression in the CMZ of the retina. Depletion of Tubgcp3 in RPCs results in monopolar spindle formation and impairs the distribution of centrioles and γ-tubulin, causing M-phase arrest and further apoptosis. In addition, depletion of Tubgcp3 results in cell proliferation defects and senescence at the peripheral CMZ. These defects led to the small eye phenotype in the *tubgcp3* mutants. To our knowledge, this is the first *in vivo* model for the functional study of Tubgcp3. Our findings also provide some clues for studying the roles of its associated complexes, γ-TuSC and γ-TuRC, in vertebrate development.

γ-TuSC, composed of Tubgcp3, Tubgcp2, and γ-tubulin, is an essential complex for microtubule nucleation and spindle assembly during mitosis. Each component of γ-TuSC is likely essential for all cell proliferation in the developing zebrafish. Recently, Pouchucq et al. (2018) reported that morpholino (MO)-mediated *tubg1*/*γ-tubulin* knockdown caused zebrafish embryo development arrest at the mid-gastrula stage. The early developmental defects are most probably attributable to the fact that MO was designed against the translation start site of *tubg1* mRNA, which blocks the translation of both the maternal and zygotic *tubg1* mRNA (Pouchucq et al., 2018). Although cell cycle defects and increased apoptosis were observed after depletion of γ-tubulin (Pouchucq et al., 2018), study on the function of γ-tubulin in later stages of development has been limited. In contrast, the *tubgcp3* mutants can survive longer than 10 days due to the maternal deposition of *tubgcp3* gene products. The maternal effect provides a possibility to study its function at relatively late developmental stages. In the *tubgcp3* mutant, the differentiated cells seem to be unaffected (Figure 3I–L). However, the CMZ exhibits obvious defects from 3 to 5 dpf (Figure 3E–H). This is associated with the relatively higher expression of *tubgcp3* in CMZ compared to other retinal regions (Figure 1L,M). In CMZ, new neurons are produced for the continuous growth of zebrafish eyes after the embryogenesis of retina completed at 60 hpf. The tissue-specific defects in CMZ are in accordance with the rapid depletion of the maternal store in the regions of high proliferation. This also correlates with the function of γ-TuSC in cell proliferation.

Despite the fact that many studies have been carried out among different organisms to understand the function of Tubgcp3 in γ-TuRC assembly and centrosome function (Geissler et al., 1996; Barbosa et al., 2000; Mikule et al., 2007; Xiong and Oakley, 2009; Farache et al., 2016; Cota et al., 2017), its roles in vertebrate development remain unclear. *In vitro* studies using human cell lines show that GCP3/Tubgcp3 is required for cell cycle progression (Mikule et al., 2007; Draberova et al., 2015; Cota et al., 2017). However, the cell cycle defects caused by GCP3 depletion are conflicting. Draberova et al. (2015) reported that the wild-type p53 U87MG cells and mutant p53 T98 cells arrested in M-phase after depletion of GCP3 using siRNA. GCP3-depleted HeLa cells also arrested in the M-phase (Cota et al., 2017). In the *tubgcp3* mutants, we found that RPCs arrested in M-phase in the CMZ of the retina (Figure 5B,D,F,G), which is consistent with previous studies. Moreover, the mitotic arrest RPCs underwent apoptosis (Figure 6A–R). However, Mikule et al. (2007) found that some GCP3-depleted cell lines, such as RPE-1, BJ-1, HME-1, and HCT-116, did not arrest in M-phase, but arrested in G1 phase in a p53-dependent manner. In our study, we found that cell proliferation was affected at the peripheral CMZ in the *tubgcp3* mutant retina. These cells might be RSCs, which failed to progress into S phase (Figure 6S–T”,Y). They did not arrest in M-phase as PH3+ cells did not accumulate in the mutant peripheral CMZ at 5 dpf (Supplementary Figure S7). It is not feasible for us to isolate RSCs for fluorescence-activated cell sorter analysis (FACS) due to the absence of available transgenic zebrafish lines expressing RSC-specific reporters in our lab. Whether depletion of Tubgcp3 in RSCs causes G1 arrest still needs further study. To study whether p53 is involved in these processes will be an important extension to the functional study of Tubgcp3. It has been reported that centrosome dysfunction could induce cellular senescence (Manning and Kumar, 2010; Schmidt et al., 2010; Hossain and Tsang, 2013). Consistent with these reports, we observed that some cells underwent senescence at the extreme periphery of CMZ in *tubgcp3* mutants. Since CMZ cells are involved in the growth of zebrafish eye, these defects together caused the small eye phenotype in the *tubgcp3* mutants.

Autosomal recessive primary microcephaly (MCPH) is a neurodevelopmental disorder characterized by markedly reduced brain size. The patients exhibited significantly reduced number of neural progenitor cells caused by cell proliferation defects, increased cell death and the disruption of the balance between symmetric and asymmetric division (Barbelanne and Tsang, 2014; Buchwalter et al., 2016; O’Neill et al., 2018). Currently, at least nine centrosome-related genes have been genetically linked to microcephaly disorders (Megraw et al., 2011; Novorol et al., 2013; Chavali et al., 2014; Morris-Rosendahl and Kaindl, 2015; Buchwalter et al., 2016). Mutations in *tubgcp4* and *tubgcp6* have been reported to be associated with MCPH (Buchwalter et al., 2016). They encode TUBGCP4 and TUBGCP6, respectively, two components of γ-TuRC (Zheng et al., 1995). In addition, the MCPH models have been established using zebrafish retinal neuroepithelium by depletion of centrosomal proteins, including STIL, ASPM, WDR62, and ODF2 (Novorol et al., 2013). STIL localizes to the procentriolar cartwheel region and plays important roles in centriole duplication (Arquint and Nigg, 2014). ASPM localizes at the spindle poles and is involved in the organization of spindle poles (Tungadi et al., 2017). WDR62, which also localizes to the spindle poles, is required for mitotic entry of neural stem cells (Ramdas Nair et al., 2016). ODF2, a mother centriole subdistal appendage protein, is indispensable for the assembly of the mother centriole (Ishikawa et al., 2005). In these models, zebrafish embryos display the MCPH phenotype with significantly reduced head and eye size (Novorol et al., 2013). After knockout of *tubgcp3* gene, the zebrafish embryos exhibit the similar MCPH phenotype (Figure 2E–G). M-phase cell cycle arrest (Figure 5A–G) and increased apoptosis are also observed (Figure 6A–R) in *tubgcp3* mutant retinae, which is consistent with these MCPH models (Novorol et al., 2013). Moreover, cell proliferation defect and senescence were also observed at the peripheral CMZ (stem cell niche) (Figure 6S–V,Y). Senescent cells were also observed in the brain of the *tubgcp3* mutants (Supplementary Figure S8). Given the MCPH phenotype observed in *tubgcp3* mutants (Figure 2E–G) and the requirement of Tubgcp3 in γ-TuRC assembly and centrosome function (Geissler et al., 1996; Barbosa et al., 2000; Mikule et al., 2007; Xiong and Oakley, 2009; Farache et al., 2016; Cota et al., 2017), we speculate that premature senescence may provide a possible new view to explain the mechanism of MCPH. Further studies to characterize the function of other centrosomal proteins will be necessary to fully understand the relationship between centrosome and microcephaly disorders.

## Ethics Statement

This study was carried out according to the Guide for the Care and Use of Laboratory Animals from the National Institutes of Health. The experimental protocol was approved by the Animal Care and Use Committee of Fudan University.

## Author Contributions

TZ conceived and directed the project. GL carried out the experiments and discovered the roles of Tubgcp3 in cell cycle progression during zebrafish retinal development. TZ, DJ, and GL prepared the figures and wrote the manuscript.

## Conflict of Interest Statement

The authors declare that the research was conducted in the absence of any commercial or financial relationships that could be construed as a potential conflict of interest.

## References

[B1] Arquint C. Nigg E. A. (2014). STIL microcephaly mutations interfere with APC/C-mediated degradation and cause centriole amplification. *Curr. Biol.* 24 351–360. 10.1016/j.cub.2013.12.016 24485834

[B2] Barbelanne M. Tsang W. Y. (2014). Molecular and cellular basis of autosomal recessive primary microcephaly. *Biomed. Res. Int.* 2014:547986. 10.1155/2014/547986 25548773 PMC4274849

[B3] Barbosa V. Yamamoto R. R. Henderson D. S. Glover D. M. (2000). Mutation of a drosophila gamma tubulin ring complex subunit encoded by discs degenerate-4 differentially disrupts centrosomal protein localization. *Genes Dev.* 14 3126–3139. 10.1101/gad.182800 11124805 PMC317135

[B4] Bornens M. (2002). Centrosome composition and microtubule anchoring mechanisms. *Curr. Opin. Cell Biol.* 14 25–34. 10.1016/S0955-0674(01)00290-3 11792541

[B5] Buchwalter R. A. Chen J. V. Zheng Y. Megraw T. L. (2016). Centrosome in cell division, development and disease. *eLS* 30 1–2.

[B6] Cerveny K. L. Cavodeassi F. Turner K. J. de Jong-Curtain T. A. Heath J. K. Wilson S. W. (2010). The zebrafish flotte lotte mutant reveals that the local retinal environment promotes the differentiation of proliferating precursors emerging from their stem cell niche. *Development* 137 2107–2115. 10.1242/dev.047753 20504962 PMC2882130

[B7] Chavali P. L. Putz M. Gergely F. (2014). Small organelle, big responsibility: the role of centrosomes in development and disease. *Philos. Trans. R. Soc. Lond. B Biol. Sci.* 369:20130468. 10.1098/rstb.2013.0468 25047622 PMC4113112

[B8] Cota R. R. Teixido-Travesa N. Ezquerra A. Eibes S. Lacasa C. Roig J. (2017). MZT1 regulates microtubule nucleation by linking gammaTuRC assembly to adapter-mediated targeting and activation. *J. Cell Sci.* 130 406–419. 10.1242/jcs.195321 27852835

[B9] Dimri G. P. Lee X. H. Basile G. Acosta M. Scott C. Roskelley C. (1995). A biomarker that identifies senescent human-cells in culture and in aging skin *In-Vivo*. *Proc. Natl. Acad. Sci. U.S.A.* 92 9363–9367. 10.1073/pnas.92.20.9363 7568133 PMC40985

[B10] Draberova E. D’Agostino L. Caracciolo V. Sladkova V. Sulimenko T. Sulimenko V. (2015). Overexpression and nucleolar localization of gamma-tubulin small complex proteins GCP2 and GCP3 in glioblastoma. *J. Neuropathol. Exp. Neurol.* 74 723–742. 10.1097/NEN.0000000000000212 26079448

[B11] Farache D. Jauneau A. Chemin C. Chartrain M. Remy M. H. Merdes A. (2016). Functional analysis of gamma-tubulin complex proteins indicates specific lateral association via their N-terminal domains. *J. Biol. Chem.* 291 23112–23125. 10.1074/jbc.M116.744862 27660388 PMC5087730

[B12] Geissler S. Pereira G. Spang A. Knop M. Soues S. Kilmartin J. (1996). The spindle pole body component Spc98p interacts with the gamma-tubulin-like Tub4p of Saccharomyces cerevisiae at the sites of microtubule attachment. *EMBO J.* 15 3899–3911. 10.1002/j.1460-2075.1996.tb00764.x 8670895 PMC452092

[B13] Gonzalez-Nunez V. Nocco V. Budd A. (2010). Characterization of drCol 15a1b: a novel component of the stem cell niche in the zebrafish retina. *Stem Cells* 28 1399–1411. 10.1002/stem.461 20549708

[B14] Hossain D. Tsang W. Y. (2013). Centrosome dysfunction and senescence: coincidence or causality? *J. Aging Sci.* 1:3 10.4172/2329-8847.1000113

[B15] Huang X. Kurose A. Tanaka T. Traganos F. Dai W. Darzynkiewicz Z. (2006). Sequential phosphorylation of Ser-10 on histone H3 and ser-139 on histone H2AX and ATM activation during premature chromosome condensation: relationship to cell-cycle phase and apoptosis. *Cytometry A* 69 222–229. 10.1002/cyto.a.20257 16528736

[B16] Ishikawa H. Kubo A. Tsukita S. Tsukita S. (2005). Odf2-deficient mother centrioles lack distal/subdistal appendages and the ability to generate primary cilia. *Nat. Cell Biol.* 7 517–524. 10.1038/ncb1251 15852003

[B17] Kellogg D. R. Moritz M. Alberts B. M. (1994). The centrosome and cellular organization. *Annu. Rev. Biochem.* 63 639–674. 10.1146/annurev.bi.63.070194.003231 7979251

[B18] Knop M. Pereira G. Geissler S. Grein K. Schiebel E. (1997). The spindle pole body component Spc97p interacts with the gamma-tubulin of Saccharomyces cerevisiae and functions in microtubule organization and spindle pole body duplication. *EMBO J.* 16 1550–1564. 10.1093/emboj/16.7.1550 9130700 PMC1169759

[B19] Kollman J. M. Polka J. K. Zelter A. Davis T. N. Agard D. A. (2010). Microtubule nucleating gamma-TuSC assembles structures with 13-fold microtubule-like symmetry. *Nature* 466 879–882. 10.1038/nature09207 20631709 PMC2921000

[B20] Li Z. Joseph N. M. Easter S. S. Jr. (2000). The morphogenesis of the zebrafish eye, including a fate map of the optic vesicle. *Dev. Dyn.* 218 175–188. 10822269 10.1002/(SICI)1097-0177(200005)218:1<175::AID-DVDY15>3.0.CO;2-K

[B21] Manning J. A. Kumar S. (2010). A potential role for NEDD1 and the centrosome in senescence of mouse embryonic fibroblasts. *Cell Death Dis.* 1:e35. 10.1038/cddis.2010.12 21364642 PMC3032305

[B22] Marcus R. C. Delaney C. L. Easter S. S. Jr. (1999). Neurogenesis in the visual system of embryonic and adult zebrafish (Danio rerio). *Vis. Neurosci.* 16 417–424. 10.1017/s095252389916303x 10349963

[B23] Masai I. Stemple D. L. Okamoto H. Wilson S. W. (2000). Midline signals regulate retinal neurogenesis in zebrafish. *Neuron* 27 251–263. 10.1016/S0896-6273(00)00034-9 10985346

[B24] Megraw T. L. Sharkey J. T. Nowakowski R. S. (2011). Cdk5rap2 exposes the centrosomal root of microcephaly syndromes. *Trends Cell Biol.* 21 470–480. 10.1016/j.tcb.2011.04.007 21632253 PMC3371655

[B25] Mikule K. Delaval B. Kaldis P. Jurcyzk A. Hergert P. Doxsey S. (2007). Loss of centrosome integrity induces p38-p53-p21-dependent G1-S arrest. *Nat. Cell Biol.* 9 160–170. 17330329 10.1038/ncb1529

[B26] Morris-Rosendahl D. J. Kaindl A. M. (2015). What next-generation sequencing (NGS) technology has enabled us to learn about primary autosomal recessive microcephaly (MCPH). *Mol. Cell. Probes* 29 271–281. 10.1016/j.mcp.2015.05.015 26050940

[B27] Novorol C. Burkhardt J. Wood K. J. Iqbal A. Roque C. Coutts N. (2013). Microcephaly models in the developing zebrafish retinal neuroepithelium point to an underlying defect in metaphase progression. *Open Biol.* 3:130065. 10.1098/rsob.130065 24153002 PMC3814721

[B28] Oakley B. R. Oakley C. E. Yoon Y. S. Jung M. K. (1990). Gamma-tubulin is a component of the spindle pole body that is essential for microtubule function in aspergillus-nidulans. *Cell* 61 1289–1301. 10.1016/0092-8674(90)90693-9 2194669

[B29] Ohnuma S. Philpott A. Wang K. Holt C. E. Harris W. A. (1999). p27Xic1, a Cdk inhibitor, promotes the determination of glial cells in xenopus retina. *Cell* 99 499–510. 10.1016/S0092-8674(00)81538-X 10589678

[B30] O’Neill R. S. Schoborg T. A. Rusan N. M. (2018). Same but different: pleiotropy in centrosome-related microcephaly. *Mol. Biol. Cell* 29 241–246. 10.1091/mbc.E17-03-0192 29382806 PMC5996963

[B31] Park H. C. Kim C. H. Bae Y. K. Yeo S. Y. Kim S. H. Hong S. K. (2000). Analysis of upstream elements in the HuC promoter leads to the establishment of transgenic zebrafish with fluorescent neurons. *Dev. Biol.* 227 279–293. 10.1006/dbio.2000.9898 11071755

[B32] Pelegri F. (2003). Maternal factors in zebrafish development. *Dev. Dyn.* 228 535–554. 10.1002/dvdy.10390 14579391

[B33] Pereira G. Knop M. Schiebel E. (1998). Spc98p directs the yeast gamma-tubulin complex into the nucleus and is subject to cell cycle-dependent phosphorylation on the nuclear side of the spindle pole body. *Mol. Biol. Cell* 9 775–793. 10.1091/mbc.9.4.775 9529377 PMC25305

[B34] Pouchucq L. Undurraga C. A. Fuentes R. Cornejo M. Allende M. L. Monasterio O. (2018). γ-Tubulin small complex formation is essential for early zebrafish embryogenesis. *Mechan. Dev.* 154 145–152. 10.1016/j.mod.2018.06.006 30426927

[B35] Pujic Z. Omori Y. Tsujikawa M. Thisse B. Thisse C. Malicki J. (2006). Reverse genetic analysis of neurogenesis in the zebrafish retina. *Dev. Biol.* 293 330–347. 10.1016/j.ydbio.2005.12.056 16603149

[B36] Ramdas Nair A. Singh P. Salvador Garcia D. Rodriguez-Crespo D. Egger B. Cabernard C. (2016). The microcephaly-associated protein Wdr62/CG7337 is required to maintain centrosome asymmetry in drosophila neuroblasts. *Cell Rep.* 14 1100–1113. 10.1016/j.celrep.2015.12.097 26804909

[B37] Rogakou E. P. Nieves-Neira W. Boon C. Pommier Y. Bonner W. M. (2000). Initiation of DNA fragmentation during apoptosis induces phosphorylation of H2AX histone at serine 139. *J. Biol. Chem.* 275 9390–9395. 10.1074/jbc.275.13.9390 10734083

[B38] Schmidt S. Schneider L. Essmann F. Cirstea I. C. Kuck F. Kletke A. (2010). The centrosomal protein TACC3 controls paclitaxel sensitivity by modulating a premature senescence program. *Oncogene* 29 6184–6192. 10.1038/onc.2010.354 20729911

[B39] Shkumatava A. Neumann C. J. (2005). Shh directs cell-cycle exit by activating p57Kip2 in the zebrafish retina. *EMBO Rep.* 6 563–569. 10.1038/sj.embor.7400416 15891769 PMC1369088

[B40] Thisse C. Thisse B. (2008). High-resolution in situ hybridization to whole-mount zebrafish embryos. *Nat. Protoc.* 3 59–69. 10.1038/nprot.2007.514 18193022

[B41] Tungadi E. A. Ito A. Kiyomitsu T. Goshima G. (2017). Human microcephaly ASPM protein is a spindle pole-focusing factor that functions redundantly with CDK5RAP2. *J. Cell. Sci.* 130 3676–3684. 10.1242/jcs.203703 28883092

[B42] Valdivia L. E. Lamb D. B. Horner W. Wierzbicki C. Tafessu A. Williams A. M. (2016). Antagonism between Gdf6a and retinoic acid pathways controls timing of retinal neurogenesis and growth of the eye in zebrafish. *Development* 143 1087–1098. 10.1242/dev.130922 26893342 PMC4852494

[B43] Vitorino M. Jusuf P. R. Maurus D. Kimura Y. Higashijima S. Harris W. A. (2009). Vsx2 in the zebrafish retina: restricted lineages through derepression. *Neural Dev.* 4:14. 10.1186/1749-8104-4-14 19344499 PMC2683830

[B44] Wan Y. Almeida A. D. Rulands S. Chalour N. Muresan L. Wu Y. (2016). The ciliary marginal zone of the zebrafish retina: clonal and time-lapse analysis of a continuously growing tissue. *Development* 143 1099–1107. 10.1242/dev.133314 26893352 PMC4852496

[B45] Wehman A. M. Staub W. Baier H. (2007). The anaphase-promoting complex is required in both dividing and quiescent cells during zebrafish development. *Dev. Biol.* 303 144–156. 10.1016/j.ydbio.2006.10.043 17141209

[B46] Wehman A. M. Staub W. Meyers J. R. Raymond P. A. Baier H. (2005). Genetic dissection of the zebrafish retinal stem-cell compartment. *Dev. Biol.* 281 53–65. 10.1016/j.ydbio.2005.02.010 15848388

[B47] Westerfield M. (2000). *The Zebrafish Book. A Guide for the Laboratory Use of Zebrafish (Danio rerio)*. Eugene, OR: University of Oregon Press.

[B48] Xiong Y. Oakley B. R. (2009). In vivo analysis of the functions of gamma-tubulin-complex proteins. *J. Cell. Sci.* 122(Pt 22), 4218–4227. 10.1242/jcs.059196 19861490 PMC2776505

[B49] Yuba-Kubo A. Kubo A. Hata M. Tsukita S. (2005). Gene knockout analysis of two gamma-tubulin isoforms in mice. *Dev. Biol.* 282 361–373. 10.1016/j.ydbio.2005.03.031 15893303

[B50] Zheng Y. Wong M. L. Alberts B. Mitchison T. (1995). Nucleation of microtubule assembly by a gamma-tubulin-containing ring complex. *Nature* 378 578–583. 10.1038/378578a0 8524390

