# Supplementary material for: Tubgcp3 Is Required for Retinal Progenitor Cell Proliferation During Zebrafish Development
Source: Front Mol Neurosci. 2019 May 24;12:126. doi: 10.3389/fnmol.2019.00126 (PMC6543929; doi:10.3389/fnmol.2019.00126)
Supplement: Supplementary file 1 [file Data_Sheet_1.pdf]

Tubgcp3 is required for retinal progenitor cell proliferation during zebrafish development

Running title: Tubgcp3 in zebrafish retinal development

Guobao Li[1,2], Daqing Jin[1,2,] *, Tao P. Zhong[1,2,] *

Supplementary Material consists of 8 Figures (Supplementary Figure 1-8).

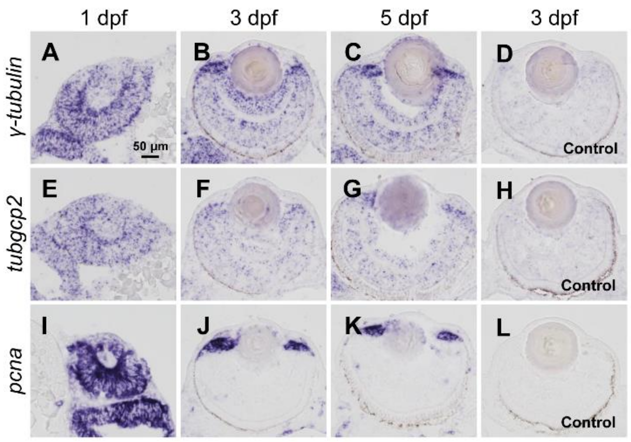

Supplementary Figure 1. Expression of *tubgcp2*, *γ-tubulin* and *pcna* during zebrafish retinal development. (**A-C**) ISH analyses showing the expression of *γ-tubulin* throughout the retina at 1 dpf and enriched at the CMZ from 3 dpf to 5 dpf (**E-G**) *tubgcp2* expression is similar to that of *γ-tubulin*. (**I-K**) *pcna* expression is throughout the retina at 1 dpf but progressively confined to the CMZ. (**D, H, L**) sense probes for *γ-tubulin, tubgcp2* and *pcna* are used as controls. Scale bars: 50 µm (**A-L**).

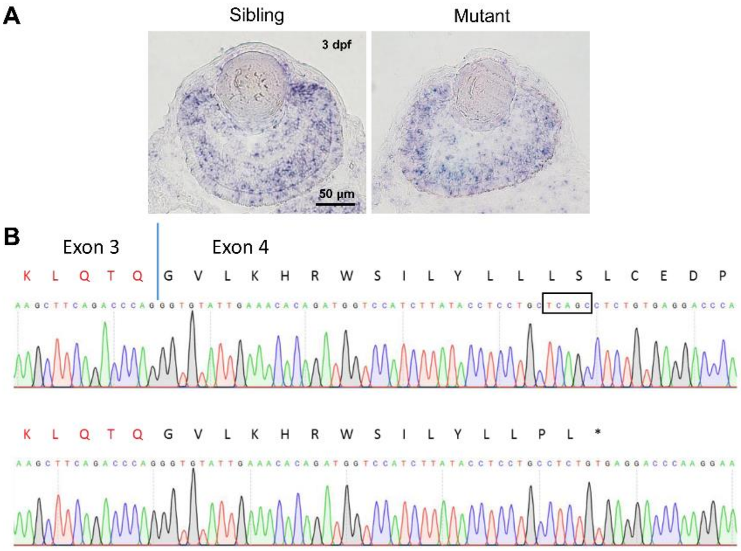

Supplementary Figure 2. The mutated *tubgcp3* mRNA escaped the nonsense-mediated decay (NMD) in the *tubgcp3* mutants. (**A**) ISH analyses show the expression of *tubgcp3* transcripts are detected in wild-type sibling and the *tubgcp3* mutant retina at 3 dpf. (**B**) Sanger sequencing of *tubgcp3* cDNA from the *tubgcp3* mutant embryos reveals a 5-nucleotide (nt) deletion (black box) in *tubgcp3* mRNA. The deletion is predicted to cause a premature stop codon (asterisk) in Tubgcp3 protein in the *tubgcp3* mutants. Scale bars: 50 μm (**A**).

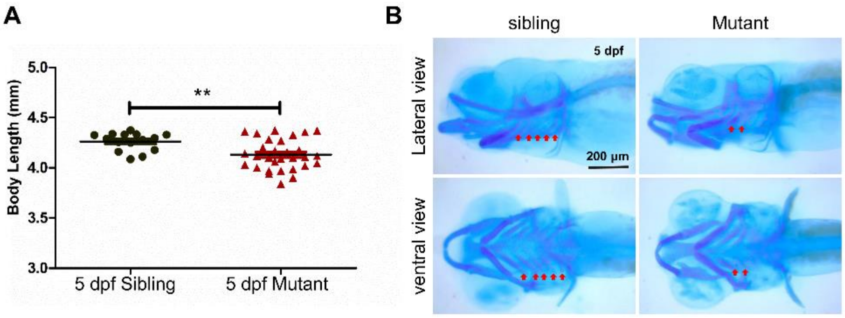

Supplementary Figure 3. The *tubgcp3* mutants exhibit short body length and branchial arch defects. (**A**) Scatter plot of body length from wild-type sibling and the *tubgcp3* mutant embryos at 5 dpf. Data are from 15 embryos for the wild-type sibling group and 30 for the *tubgcp3* mutant group. Student's t-test: $^{**}P<0.01$. (**B**) Alcian blue staining for cartilage in 5 dpf wild-type sibling and the *tubgcp3* mutant embryos. Arrows indicate the positions of branchial arches. Scale bars: 200 µm (**B**).

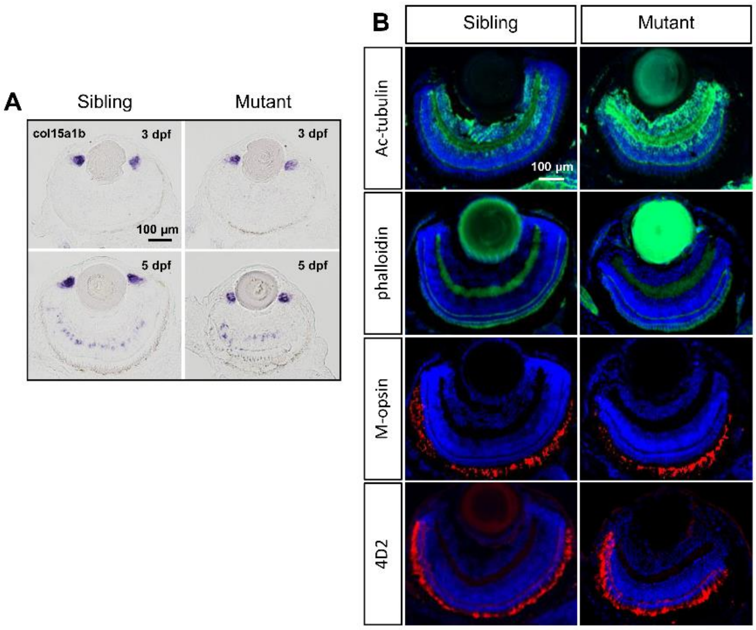

Supplementary Figure 4. The *tubgcp3* mutants exhibit normal retinal cell types and retinal laminar structures. (**A**) *col15a1b* (RSCs marker) is expressed in wild-type sibling and the *tubgcp3* mutant CMZ. (**B**) Microscopy images of several retinal cell types and laminar structures in wild-type siblings and the *tubgcp3* mutants at 5 dpf. The *tubgcp3* mutants exhibit normal cones (labeled with anti-M-opsin for cone outer segments), rods (labeled with anti-4D2 for rod outer segments) and cilia in photoreceptor cells (labeled with anti-acetylated α-tubulin). The inner segment, inter plexiform layer (IPL), outer limiting membrane (OLM) and outer plexiform layer (OPL) (labeled with phalloidin) are normal in the *tubgcp3* mutant retinae. The nuclei are counterstained with DAPI (blue). Scale bars: 100 µm (**A,B**).

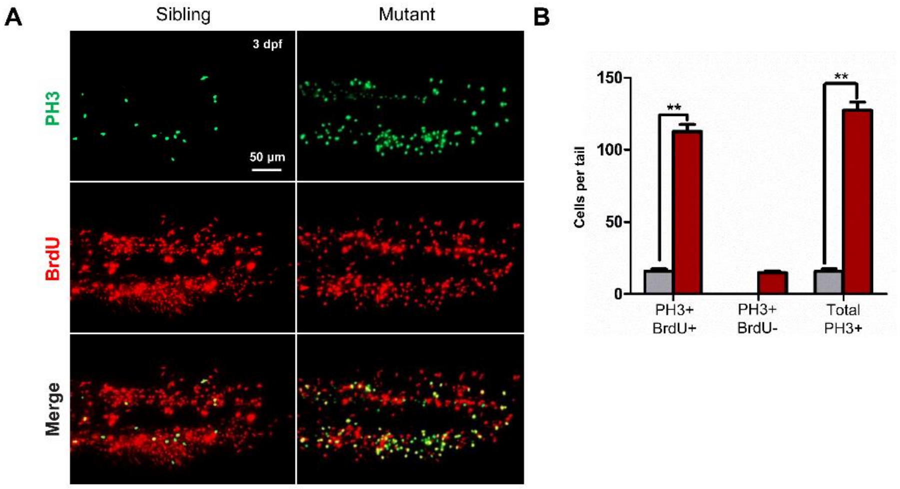

Supplementary Figure 5. Depletion of Tubgcp3 causes cell cycle arrest in M-phase in the *tubgcp3* mutant tails. (**A**) Immunostaining analysis of cell proliferation in zebrafish tails at 3 dpf using anti-BrdU (red) and anti-PH3 (green) antibodies. Zebrafish embryos are treated with BrdU from 66 hpf to 72 hpf for the double staining. The immunostaining results displaying significantly increased PH3+ cells in the *tubgcp3* mutant tails compared to wild-type siblings. Note that PH3+ BrdU- cells are observed in the *tubgcp3* mutants but absent in wild-type siblings. (**B**) Bar charts depicting quantification of BrdU- and PH3-labelded cells in wild-type sibling and the *tubgcp3* mutant tails. Data are mean + SEM from 16 embryos for each group. Student's t-test: **P<0.01. Scale bars: 50 µm (**A**).

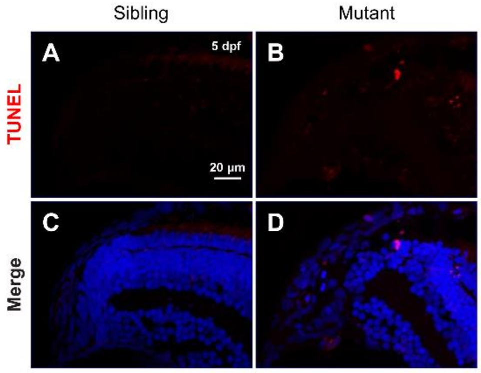

Supplementary Figure 6. Loss of Tubgcp3 in RSCs does not induce cell death. (A-D) TUNEL assay of 5 dpf eye sections exhibiting no TUNEL+ cells in the location of RSCs in the *tubgcp3* mutant retina. Scale bars: 20 µm.

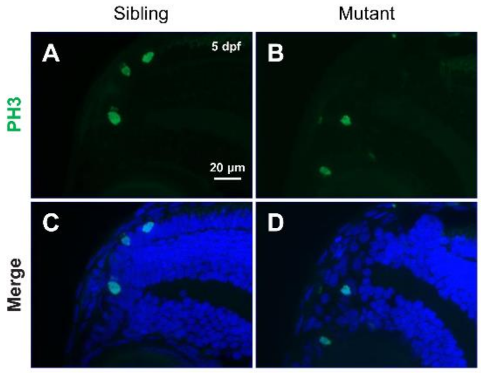

Supplementary Figure 7. Loss of Tubgcp3 in RSCs does not cause mitotic arrest. (A-D) Immunostaining analyses showing the expression of PH3 (a mitosis marker) in the *tubgcp3* mutant and wild-type sibling retina at 5 dpf. Scale bars: 20 µm.

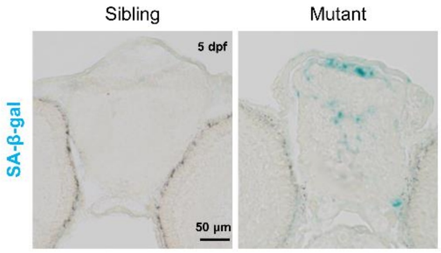

Supplementary Figure 8. Depletion of Tubgcp3 induces senescence in the *tubgcp3* mutant brains. (**A, B**) SA-β-gal staining of 5 dpf head cryosections displaying obviously increased SA-β-gal expression in the *tubgcp3* mutants compared to the wild-type siblings. Scale bars: 50 μm.
